# Supplementary material for: A wheat CC-NBS-LRR protein Ym1 confers WYMV resistance by recognizing viral coat protein
Source: Nat Commun. 2025 Apr 16;16:3630. doi: 10.1038/s41467-025-58816-0 (PMC12003722; doi:10.1038/s41467-025-58816-0)
Supplement: Supplementary file 1 — Supplementary Information [file 41467_2025_58816_MOESM1_ESM.pdf]

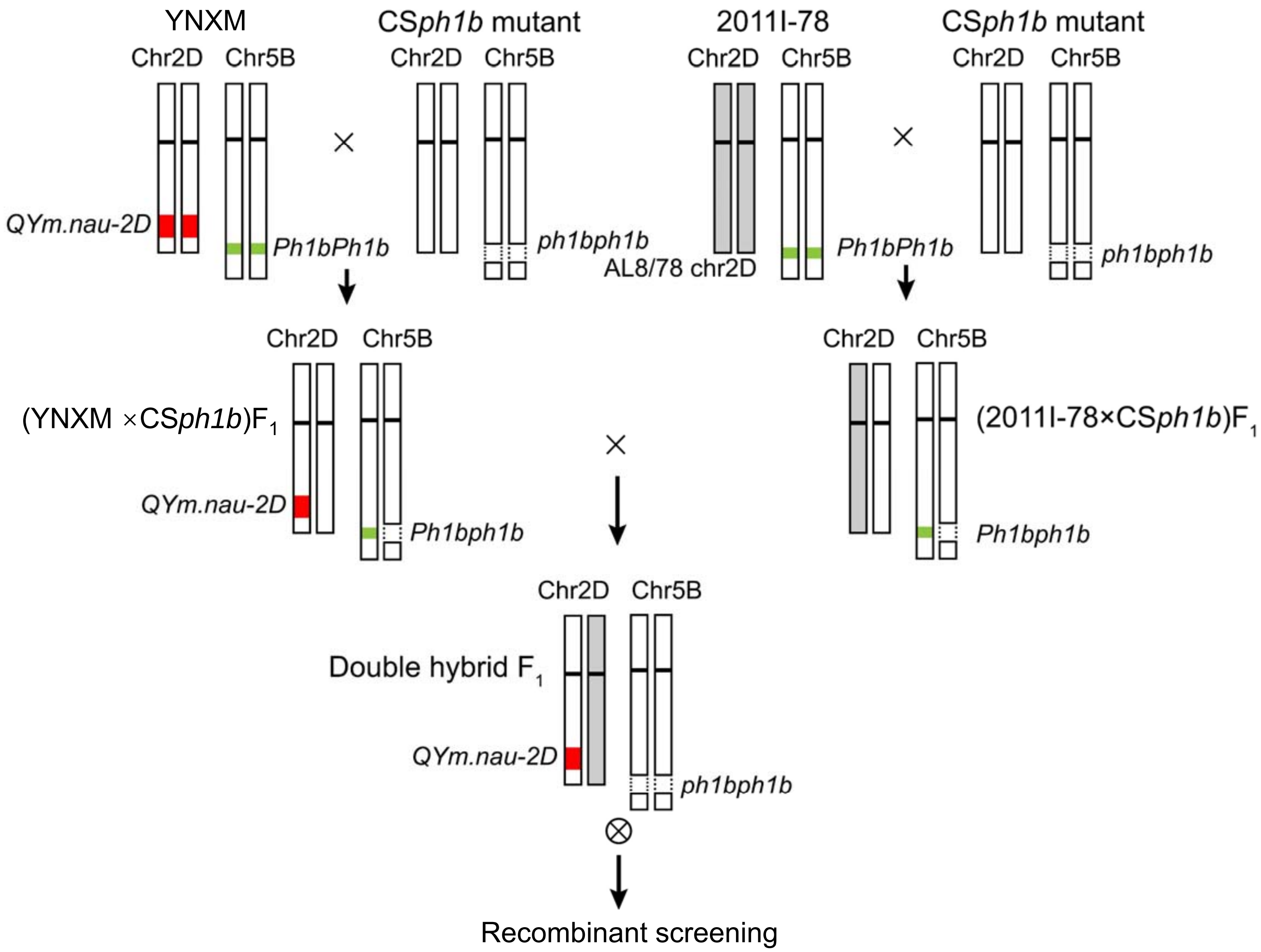


**Supplementary Figure 1 Scheme illustration showing the crosses for introducing the CS*ph1b* gene to develop segregating population used for inducing recombination of the target region**

Black frames represent chromosomes and the black line in the middle represents the centromere; red boxes represent the *QYm.nau-2D* region; green boxes represent the *Ph1b* gene region; the dotted boxes represent the *ph1b* fragment deletion

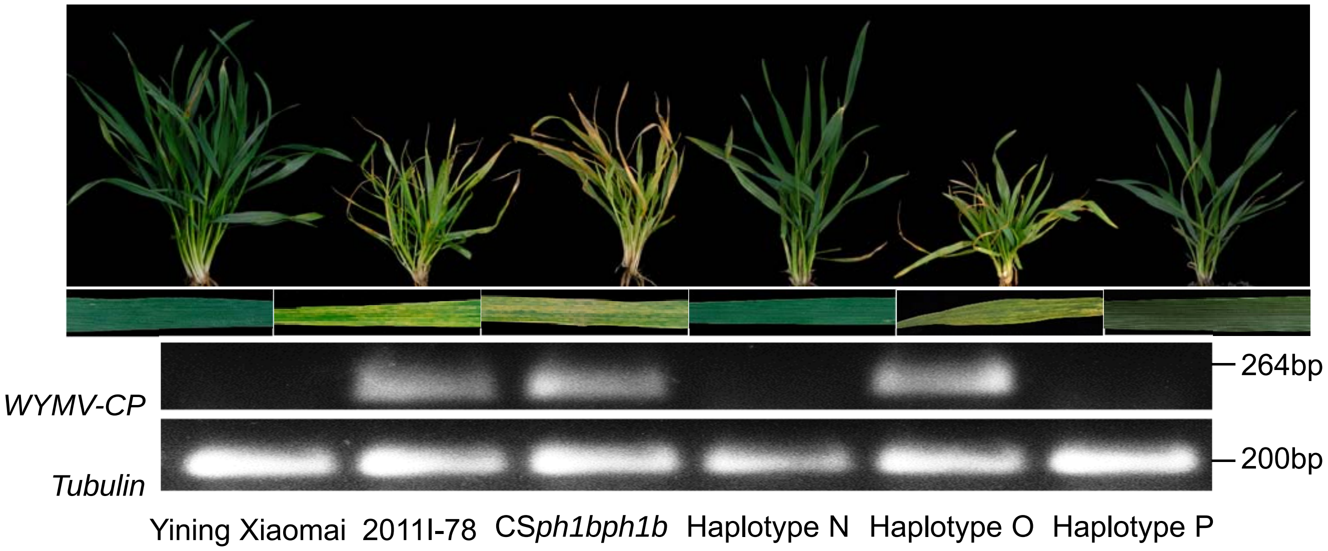


**Supplementary Figure 2 WYMV resistance evaluation of the identified three recombinants**
Upper and middle panels: WYMV symptoms of seedlings and leaves of Yining Xiaomai, 2011I-78, CS*ph1b* mutant and three recombinants. Lower panel: RT-PCR for viral *CP* abundance quantification, using wheat *Tubulin* as internal reference control. Bar=5 cm.

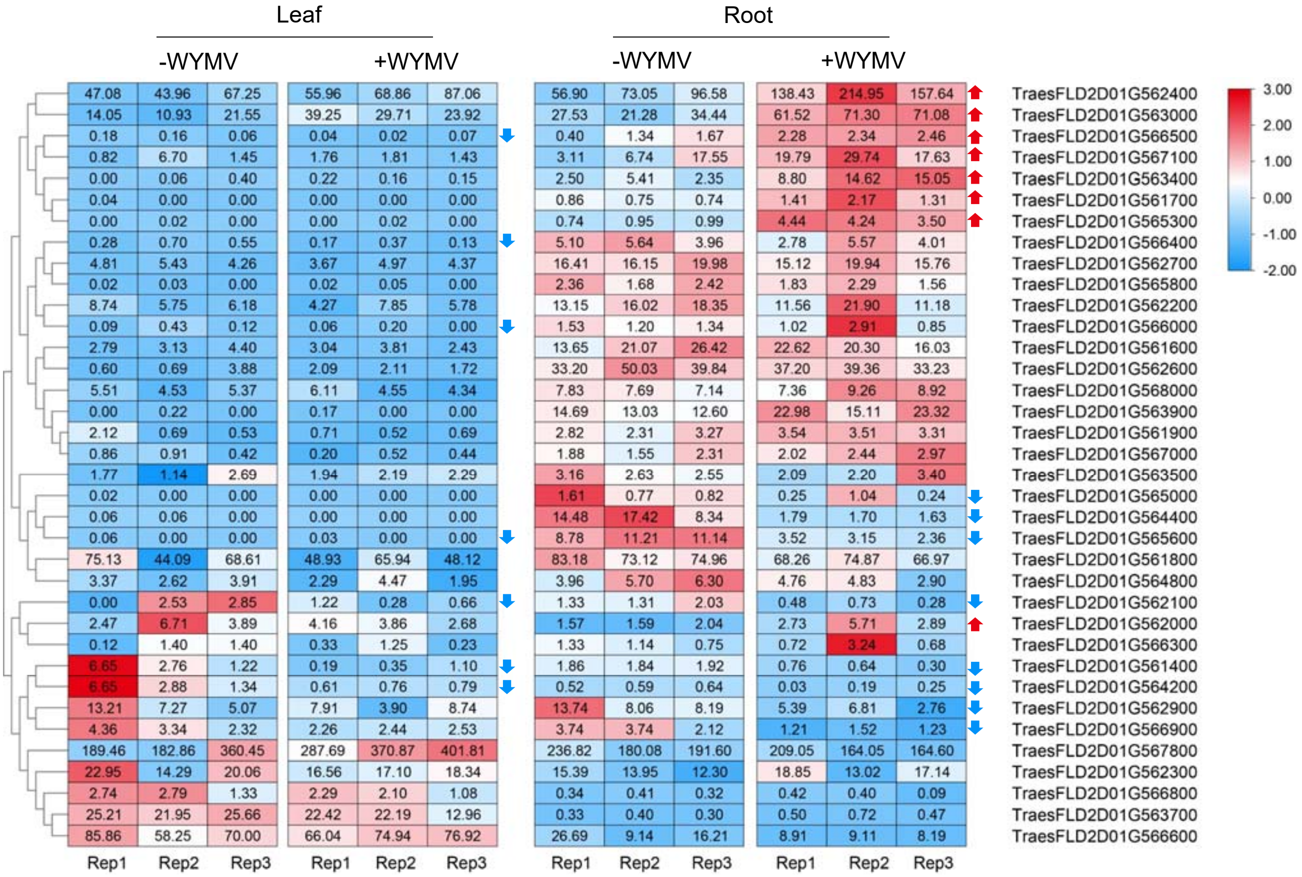


**Supplementary Figure 3 Transcriptome analysis of virus-free (-WYMV) and WYMV infected (+WYMV) root and leaf tissues of YNXM**

Heatmap of differentially expressed genes localized in the *QYm.nau-2D* mapping region revealed by TPM of RNA-seq. Three replicates were indicated as Rep1, Rep2, and Rep3. Up-arrows in red indicate up-regulated genes, down-arrows in blue indicate down-regulated genes. The asterisks show the root specific expressed DEG uniquely present in WYMV resistant Fielder genome, absent in CS genome.

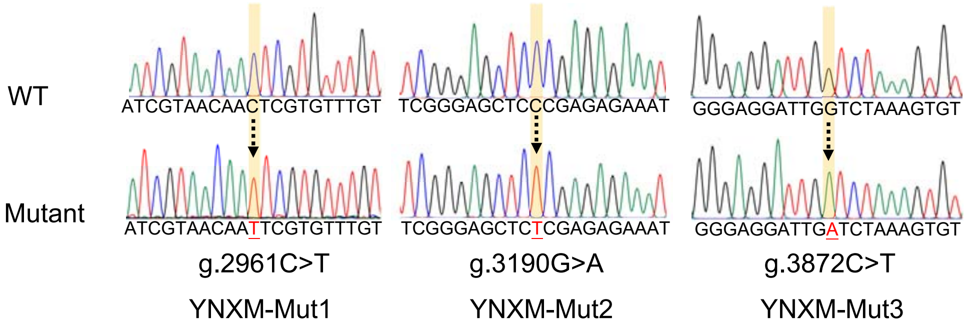


**Supplementary Figure 4 Sanger sequencing shows the polymorphisms between the wild type (WT) and three EMS mutants, YNXM-Mut1, YNXM-Mut2, and YNXM-Mut3**
The mutated nucleotides are underlined and highlighted in red.

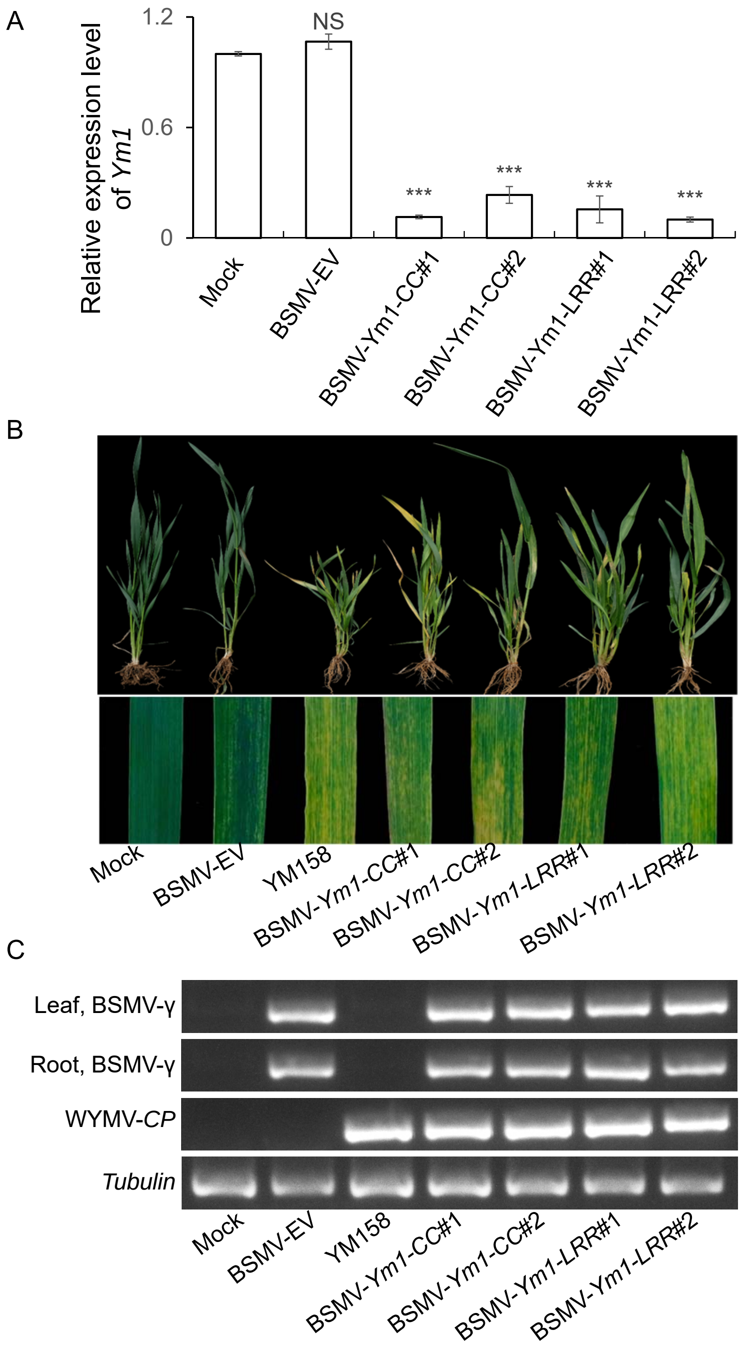


**Supplementary Figure 5 Functional analysis of *Ym1* in WYMV resistance by barley stripe mosaic virus (BSMV)-induced gene silencing (VIGS)**

A: Relative expression level of *Ym1* by qRT-PCR in roots of YNXM growing in WYMV nursery. Each data is presented as means ± SD (n=3 technical replicates). ***P<0.001, **P<0.01, *P<0.05 based on two-tailed t-tests. n.s.: no significance. B: The symptom of whole plants (upper) and leaves (lower) of the *Ym1* silenced YNXM in the WYMV nursery. Mock: no inoculation; BSMV: BSMV inoculated plants; *Ym1-CC* and *Ym1-LRR* indicate the silencing sequencing targeting the CC or LRR domains of *Ym1*. 1# and 2# are two representative plants. C: RT-PCR showing the transcripts of *BSMV-γ* in the leaves and roots, WYMV *CP* gene in leaves, using wheat *Tubulin* gene as internal reference control.

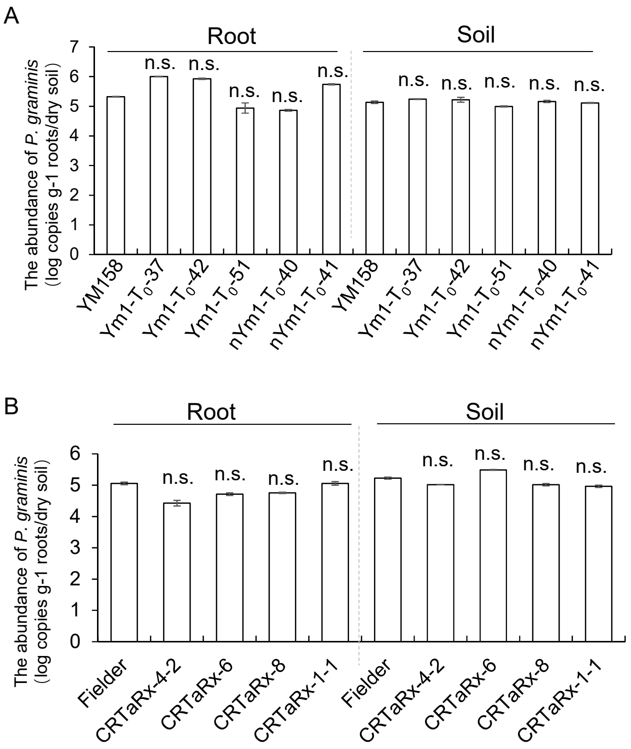


**Supplementary Figure 6 *P. graminis* fungal abundance analysis in different genotypes**

A: q-PCR of *P. graminis* abundance in the roots and soils of YM158 and transgenic plants overexpressing *Ym1* revealed with. Data are presented as means ± SD (n=3 technical replicates). n.s.: no significant in two-tailed t-tests. B: q-PCR of *P. graminis* abundance in the roots and soil of Fielder and its susceptible CR*Ym1* lines revealed with q-PCR. Data are presented as means ± SD (n=3 technical replicates). n.s.: no significant in two-tailed t-tests.

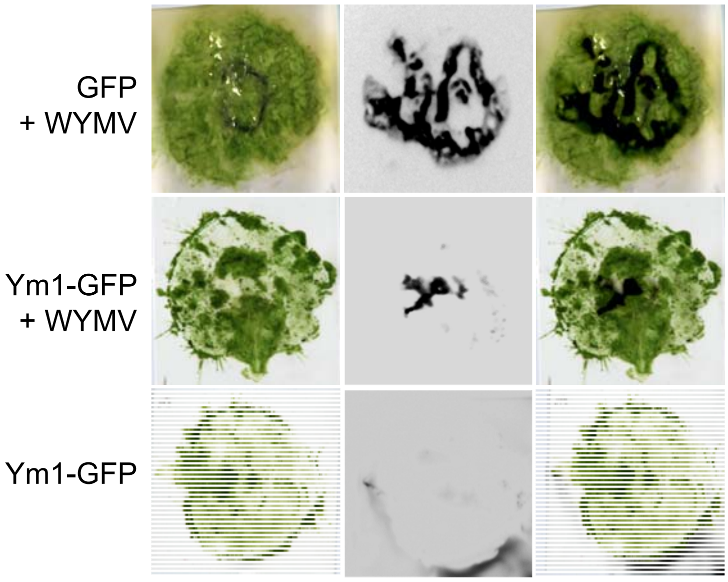


**Supplementary Figure 7   WYMV transmission experiment in *N. benthamiana* leaves**
Upper panel: co-expression of GFP empty vector and WYMV infectious clones mediated by Agrobacterium; Middle panel: co-expression of Ym1-GFP construct and WYMV infectious clones mediated by Agrobacterium; Lower panel: Expression of Ym1-GFP construct mediated by Agrobacterium.

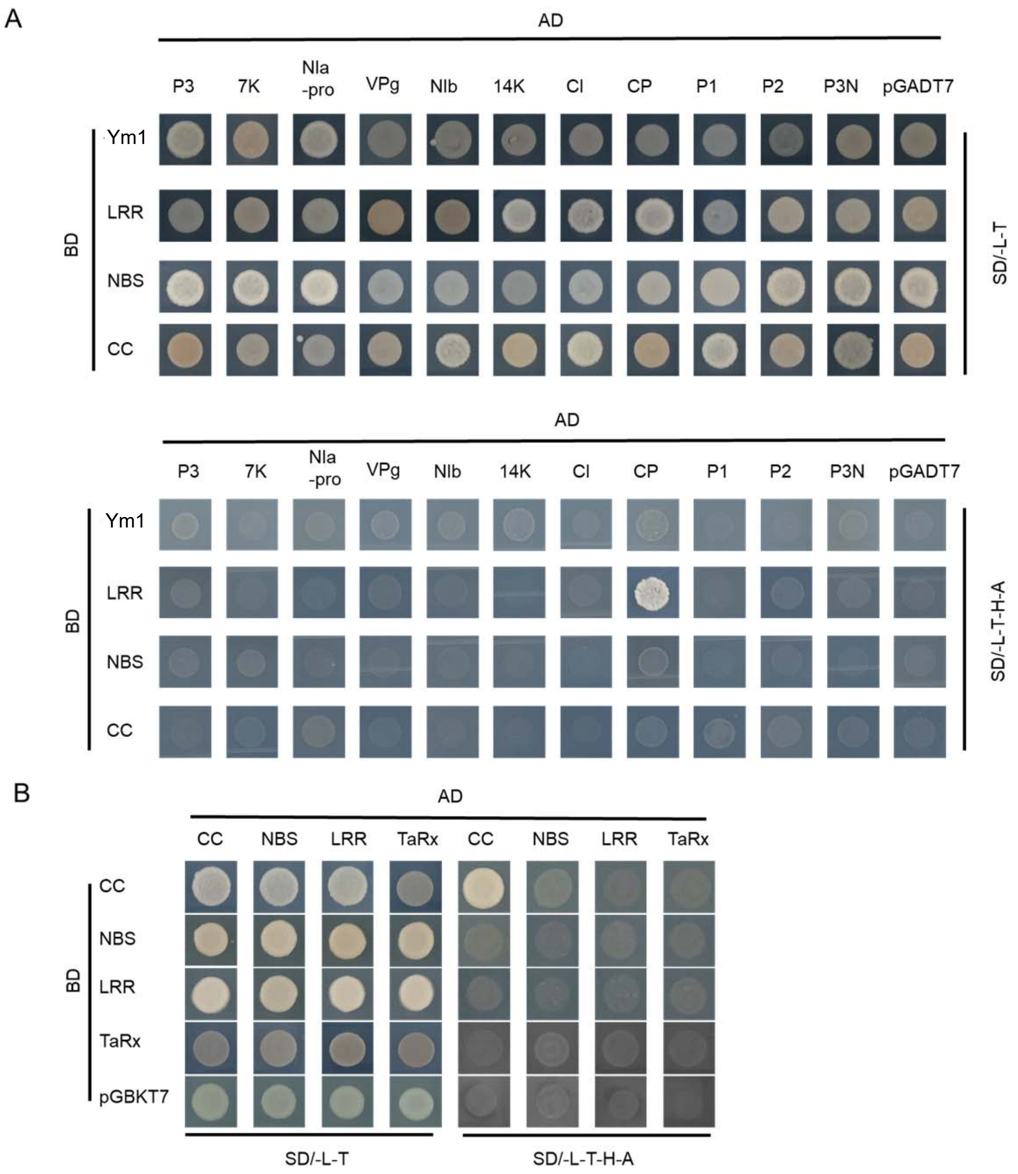


**Supplementary Figure 8 Intermolecular and intramolecular interaction analysis by yeast two-hybrid (Y2H) assays**

**A:** Y2H of 11 WYMV proteins with Ym1 and its functional domains. The whole length Ym1 and sequences of its truncated domains were fused to the Gal4 DNA-binding domain (BD); the WYMV-CP was fused to the Gal4 activation domain (AD). **B:** Intramolecular interaction among full length Ym1 and its different domains. The whole length Ym1 and three individual domains were fused to the Gal4 AD and BD, respectively. The yeast co-transformed with BD and AD constructs were plated onto synthetic dropout (SD) media lacking Leu and Trp (upper) and SD media lacking Ade, His, Leu, and Trp (lower). Images were taken at 3 days after incubation.

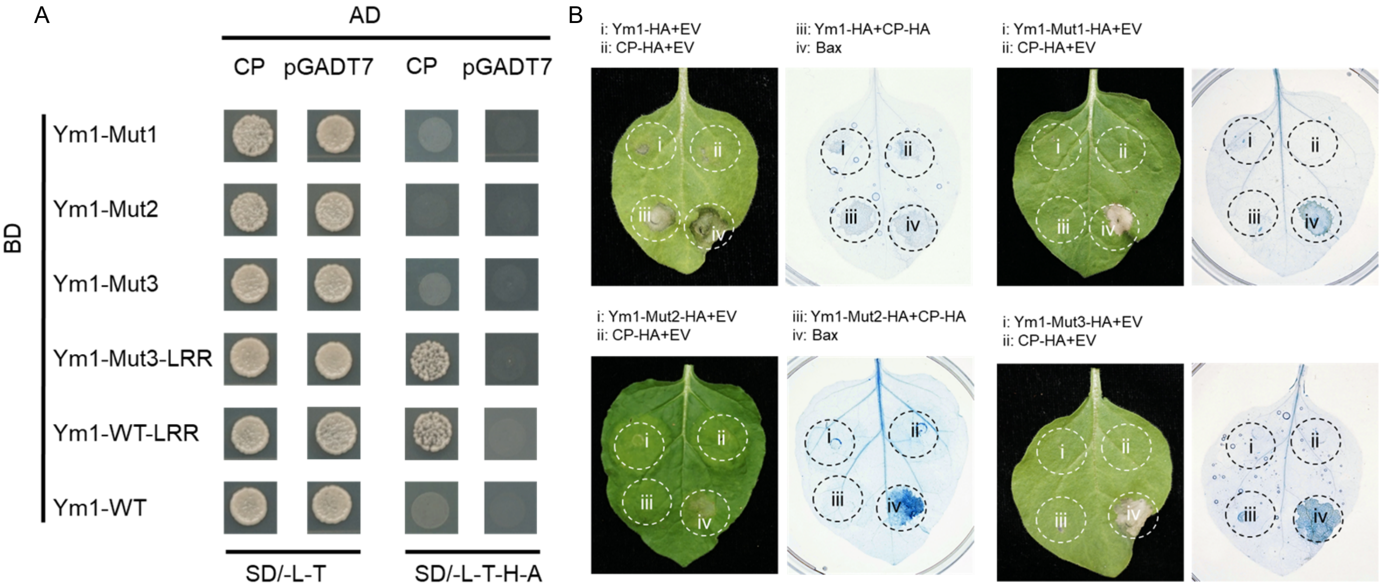


**Supplementary Figure 9 Interaction between CP and Ym1 alleles and their interactive effects**

A: Yeast two hybridization assays between CP and Ym1 alleles from three mutants. The Ym1 and its alleles sequences were fused to the Gal4 DNA-binding domain (BD); the WYMV coat protein (CP) was fused to the Gal4 activation domain (AD). The yeast co-transformed with BD and AD constructs were plated onto synthetic dropout (SD) media lacking Leu and Trp (left) and SD media lacking Ade, His, Leu, and Trp (right). Images were taken at 3 d after incubation.

B: Cell death observations of tobacco leaves after co-infiltrating *Ym1* or its alleles in three mutants and *CP* constructs.

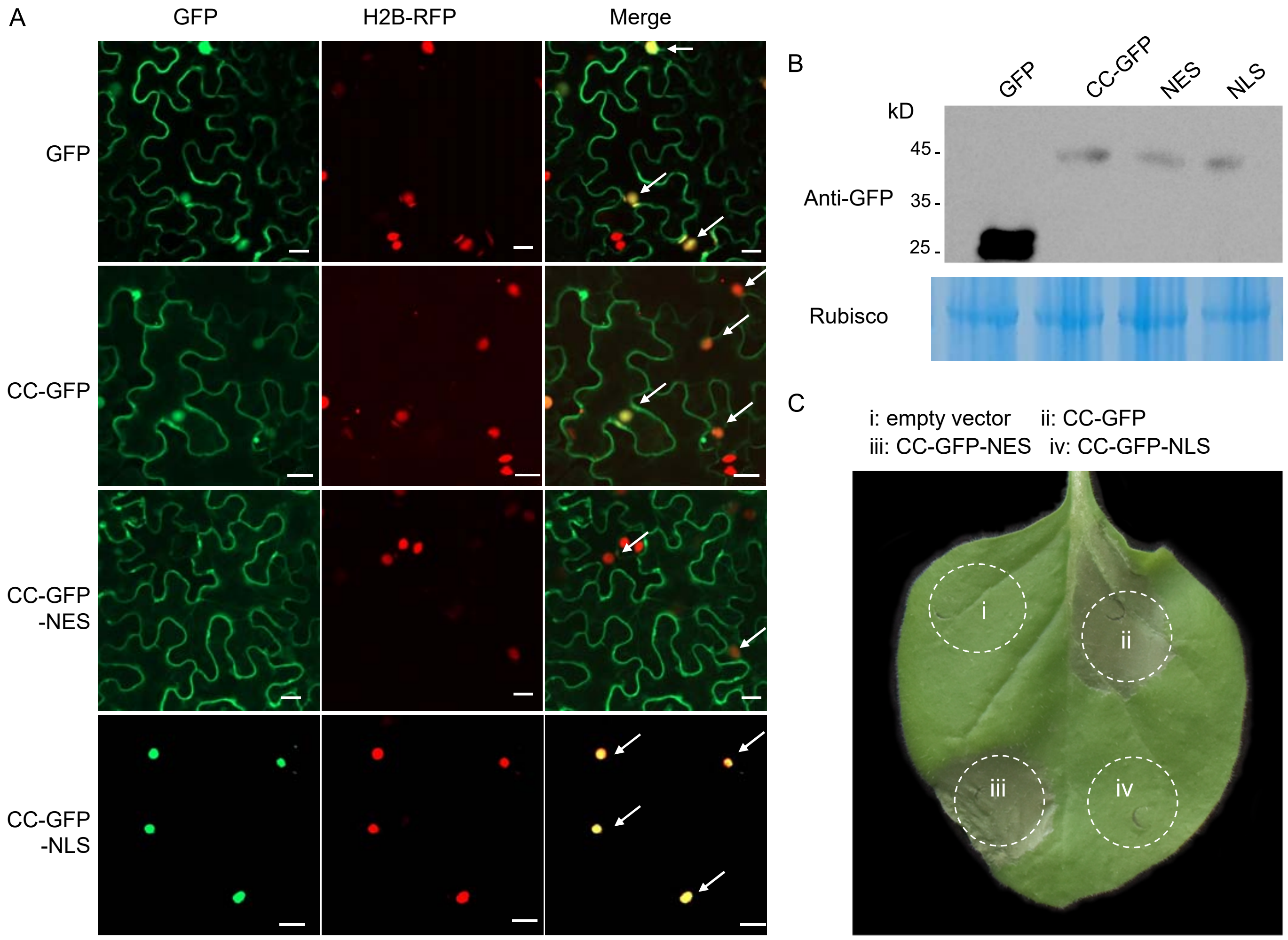


**Supplementary Figure 10 Subcellular localization analysis and assessment of cell death-inducing activity**

A: Subcellular localization of the Ym1 CC domain (CC-GFP) and CC fused with nucleus exporting (CC-GFP-NES) and localization (CC-GFP-NLS) sequences. GFP and the fusion proteins, CC-GFP, CC-GFP-NLS, and CC-GFP-NES, were expressed in *N. benthamiana* leaves by Agro-infiltration. The signals were observed under a confocal microscope at 2 dpi. Histone 2B (H2B-RFP) was used as nucleus-localized marker protein. Bars=25 μm.

B: Tissues from infiltrated sectors were sampled at 2 dpi and total protein extracts were subjected to IB with anti-GFP antibodies. CBB-stained Rubisco indicated the equal loading of different protein samples.

C: Analysis of cell death-inducing activity of the Ym1 CC domain fusion proteins. The cell death triggered by each fusion protein was scored at 4 dpi (leaf panel). A thumbnail diagram indicates the positioning of each expressed fusion protein.

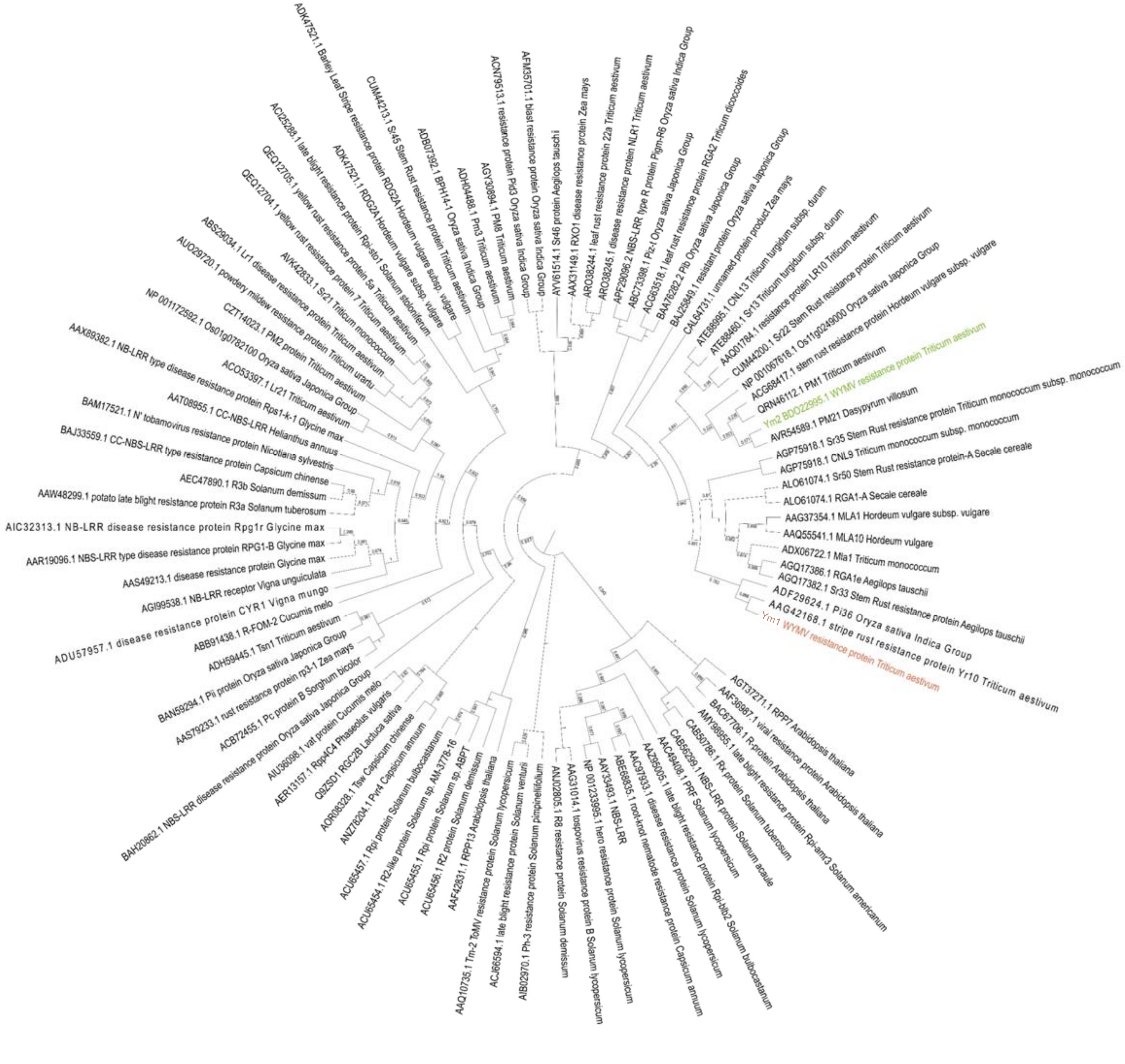


**Supplementary Figure 11 Phylogeny of 76 polypeptide products of NBS-LRR genes in *Triticum* spp. and related genera**

*Ym1* gene was highlighted in red, and another cloned WYMV-resistant gene *Ym2* was highlighted in green.

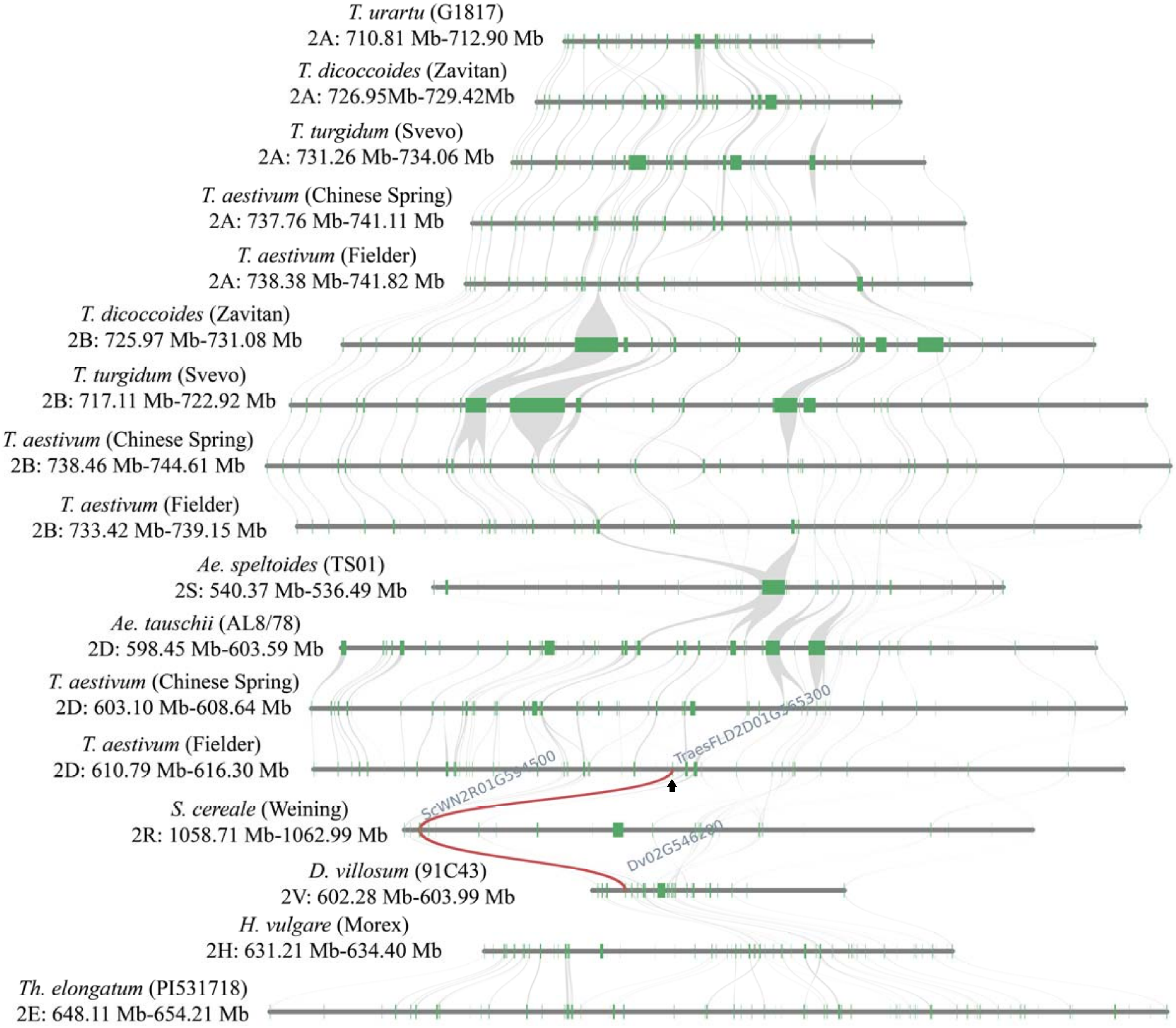


**Supplementary Figure 12 Analysis of *Ym1* homologous genes in different species or sub-genomes**
Microsynteny of *Ym1* locus on homoeologous group 2 chromosomes from multiple Triticeae species including *T. urartu*, two subgenomes of *T. dicoccoides* and *T. turgidum*, three subgenomes of Chinese Spring and Fielder, *Ae. speltoides*, *Ae. tauschii*, *S.cereale*, *D. villosum*, *H. vulgare* and *Th. elongatum*. The collinear SSP genes and the syntenic flanking genes are connected by colored and gray lines, respectively. The arrow indicates the *Ym1* genes resided in the 2D chromosome of Fielder.

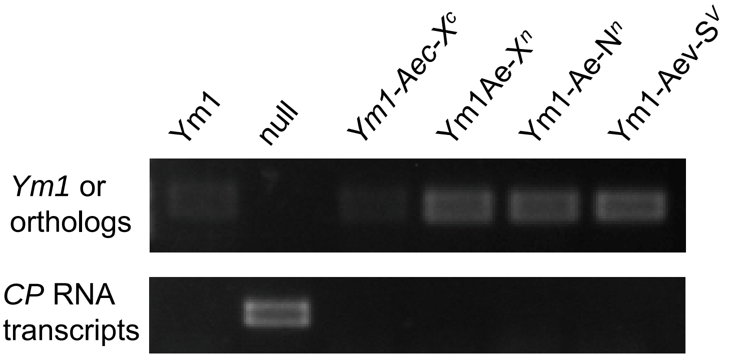


**Supplementary Figure 13 The expression of *Ym1* and orthologs of *Ym1* in roots (upper) and detection of WYMV *Coat Protein* (*CP*) RNA abundance (lower) in leaves after artificial inoculation of WYMV infectious clones**
