# Supplementary material for: A wheat CC-NBS-LRR protein Ym1 confers WYMV resistance by recognizing viral coat protein
Source: Nat Commun. 2025 Apr 16;16:3630. doi: 10.1038/s41467-025-58816-0 (PMC12003722; doi:10.1038/s41467-025-58816-0)
Supplement: Supplementary file 2 — Description of Additional Supplementary Files [file 41467_2025_58816_MOESM2_ESM.pdf]

<u>Description of Additional Supplementary Files</u>

Supplementary Data 1: Primers used in this study

Supplementary Data 2: Gene syntenic analysis between chromosome 2D of Fielder and Chinese Spring at the finally mapped region of *QYm.nau-2D*

Supplementary Data 3: SNPs calling of Yining Xiaomai mutants

Supplementary Data 4: The list of 92 CC-NBS-LRR-type NLR proteins for phylogenetic analysis

Supplementary Data 5: *Aegilops* species used in this study

Supplementary Data 6: Probes used in RNA *in situ* hybridization
