# Supplementary material for: A wheat CC-NBS-LRR protein Ym1 confers WYMV resistance by recognizing viral coat protein
Source: Nat Commun. 2025 Apr 16;16:3630. doi: 10.1038/s41467-025-58816-0 (PMC12003722; doi:10.1038/s41467-025-58816-0)
Supplement: Supplementary file 4 — Reporting Summary [file 41467_2025_58816_MOESM4_ESM.pdf]

nature portfolio

Corresponding author(s): DAPR, NCOMMS-24-58919A

Last updated by author(s): Jan 20, 2025

Reporting Summary

Nature Portfolio wishes to improve the reproducibility of the work that we publish. This form provides structure for consistency and transparency in reporting. For further information on Nature Portfolio policies, see our Editorial Policies and the Editorial Policy Checklist.

Statistics

For all statistical analyses, confirm that the following items are present in the figure legend, table legend, main text, or Methods section.

n/a	Confirmed	
☐	☒	The exact sample size (*n*) for each experimental group/condition, given as a discrete number and unit of measurement
☐	☒	A statement on whether measurements were taken from distinct samples or whether the same sample was measured repeatedly
☐	☒	The statistical test(s) used AND whether they are one- or two-sided<br>*Only common tests should be described solely by name; describe more complex techniques in the Methods section.*
☒	☐	A description of all covariates tested
☒	☐	A description of any assumptions or corrections, such as tests of normality and adjustment for multiple comparisons
☒	☐	A full description of the statistical parameters including central tendency (e.g. means) or other basic estimates (e.g. regression coefficient) AND variation (e.g. standard deviation) or associated estimates of uncertainty (e.g. confidence intervals)
☐	☒	For null hypothesis testing, the test statistic (e.g. *F*, *t*, *r*) with confidence intervals, effect sizes, degrees of freedom and *P* value noted<br>*Give P values as exact values whenever suitable.*
☒	☐	For Bayesian analysis, information on the choice of priors and Markov chain Monte Carlo settings
☒	☐	For hierarchical and complex designs, identification of the appropriate level for tests and full reporting of outcomes
☒	☐	Estimates of effect sizes (e.g. Cohen's *d*, Pearson's *r*), indicating how they were calculated

Our web collection on statistics for biologists contains articles on many of the points above.

Software and code

Policy information about availability of computer code

Data collection	Not applicable.
Data analysis	Not applicable.

For manuscripts utilizing custom algorithms or software that are central to the research but not yet described in published literature, software must be made available to editors and reviewers. We strongly encourage code deposition in a community repository (e.g. GitHub). See the Nature Portfolio guidelines for submitting code & software for further information.

Data

Policy information about availability of data

All manuscripts must include a data availability statement. This statement should provide the following information, where applicable:

- Accession codes, unique identifiers, or web links for publicly available datasets
- A description of any restrictions on data availability
- For clinical datasets or third party data, please ensure that the statement adheres to our policy

The nucleotide sequences of Ym1 and its coding protein were obtained from the GenBank database: PP909815. The transcriptomic and resequencing data generated in this study have been deposited in the Genome Sequence Archive (Genomics, Proteomics & Bioinformatics 2021) in National Genomics Data Center (Nucleic Acids Res 2022), China National Center for Bioinformation/Beijing Institute of Genomics, Chinese Academy of Sciences under accession code GSA: CRA023357, CRA023440 that are publicly accessible at https://ngdc.cncb.ac.cn/gsa. Phenotype data generated or analyzed during this study is included in this

published article (and its supplementary files). Genotype data are available from the corresponding author upon reasonable request. The source data underlying Fig.1-5, as well as Supplementary Fig. 5 and 6 are provided as a Source Data file. Source data are provided with this paper.

Research involving human participants, their data, or biological material

Policy information about studies with human participants or human data. See also policy information about sex, gender (identity/presentation), and sexual orientation and race, ethnicity and racism.

Reporting on sex and gender	Not applicable.
Reporting on race, ethnicity, or other socially relevant groupings	Not applicable.
Population characteristics	Not applicable.
Recruitment	Not applicable.
Ethics oversight	Not applicable.

Note that full information on the approval of the study protocol must also be provided in the manuscript.

Field-specific reporting

Please select the one below that is the best fit for your research. If you are not sure, read the appropriate sections before making your selection.

☒ Life sciences ☐ Behavioural & social sciences ☐ Ecological, evolutionary & environmental sciences

For a reference copy of the document with all sections, see nature.com/documents/nr-reporting-summary-flat.pdf

Life sciences study design

All studies must disclose on these points even when the disclosure is negative.

Sample size	We used 326 BC1F2 individuals heterozygous for QYm.nau-2D locus for recombinant screening. No sample-size calculation is needed, as we successfully screened three recombinant and narrow our target genomic region from 11.2 Mb to 5.6 Mb.<br>A diagnostic marker TaRx-DM developed based on the cloning of TaRx was tested using a diversity panel of 372 wheat varieties. The specific amplicons were only detected in 106 WYMV resistant varieties which carry the locus QYm.nau-2D. This confirms the effectiveness of TaRx-DM marker for tracing TaRx.
Data exclusions	No data was excluded from the analyzes
Replication	Our results obtained are replicated for at least three times.
Randomization	Not applicable.
Blinding	All the investigators were blinded to group allocation.

Reporting for specific materials, systems and methods

We require information from authors about some types of materials, experimental systems and methods used in many studies. Here, indicate whether each material, system or method listed is relevant to your study. If you are not sure if a list item applies to your research, read the appropriate section before selecting a response.

Materials & experimental systems

n/a	Involved in the study	
☐	☒	Antibodies
☒	☐	Eukaryotic cell lines
☒	☐	Palaeontology and archaeology
☒	☐	Animals and other organisms
☒	☐	Clinical data
☒	☐	Dual use research of concern
☐	☒	Plants

Methods

n/a	Involved in the study	
☒	☐	ChIP-seq
☒	☐	Flow cytometry
☒	☐	MRI-based neuroimaging

Antibodies

Antibodies used

1.Actin monoclonal antibody (Abmart, Shanghai, China, M20009).
2. HA and GFP monoclonal antibody (TransGen, Biotech, Beijing, China, HT301-01 and HT801-01).
3.HA and GFP polyclonal antibody (Abmart, Shanghai, China, PA9002 and P30010).
4.Histone H3 monoclonal antibody (HUABIO, Hangzhou, China, EM30605).
5.Anti-Mouse and Rabbit (Abbkine Scientific co., Ltd., California, USA, A21010 and A21020).

Validation

All antibodies are commercially available and validated in the literature as cited on the manufacturer's websites as well as by the datasheets they provide. Antibody validation and validation criteria are available on the following websites:
HA monoclonal antibody: https://www.ab-mart.com.cn/page.aspx?node=%2059%20&id=%20963
GFP monoclonal antibody: https://www.ab-mart.com.cn/page.aspx?node=%2059%20&id=%20971
Actin monoclonal antibody: https://www.ab-mart.com.cn/page.aspx?node=%2059%20&id=%20985
HA polyclonal antibody: https://www.ab-mart.com.cn/page.aspx?node=%2059%20&id=%2053800
GFP polyclonal antibody: https://www.ab-mart.com.cn/page.aspx?node=%2059%20&id=%20977
Histone H3 monoclonal antibody: http://www.huabio.cn/product/Histone-H3-antibody-EM30605
Anti-Mouse: https://www.abbkine.com/product/hrp-goat-anti-mouse-igg-a21010
Anti-Rabbit: https://www.abbkine.com/product/hrp-goat-anti-rabbit-igg-a21020

Dual use research of concern

Policy information about dual use research of concern

Hazards

Could the accidental, deliberate or reckless misuse of agents or technologies generated in the work, or the application of information presented in the manuscript, pose a threat to:

No	Yes	
☒	☐	Public health
☒	☐	National security
☒	☐	Crops and/or livestock
☒	☐	Ecosystems
☒	☐	Any other significant area

Experiments of concern

Does the work involve any of these experiments of concern:

No	Yes	
☒	☐	Demonstrate how to render a vaccine ineffective
☒	☐	Confer resistance to therapeutically useful antibiotics or antiviral agents
☒	☐	Enhance the virulence of a pathogen or render a nonpathogen virulent
☒	☐	Increase transmissibility of a pathogen
☒	☐	Alter the host range of a pathogen
☒	☐	Enable evasion of diagnostic/detection modalities
☒	☐	Enable the weaponization of a biological agent or toxin
☒	☐	Any other potentially harmful combination of experiments and agents

Plants

Seed stocks

The wheat varieties include Yining Xiaomai,Chinese Spring, Chinese Spring Ph1b mutant (CSph1b), Fielder, Yangmai 158. The 2011I-78 is a Chinese Spring substitution line, in which Chinese Spring chromosome 2D is substituted by Aegilops tauschii 2D. 2011I-78 were kindly provided by Dr. J. Dvorak of the University of California, Davis, USA. Plant materials used in this study also includes 124 accessions of 30 Aegilops species, in which 109 lines were provided by the USDA National Plant Germplasm System (NPGS), and 11 were provided by the Chinese Crop Germplasm Resources Information System (CGRIS). Detailed Aegilops species information is provided in Supplementary Table 4. A panel of core collection germplasm was described in our previous paper (Chen et al., 2023, https://doi.org/10.1007/s00122-023-04286-1).

Novel plant genotypes

TaRx transgenic plants are obtained by A. tumefaciens-mediated transformation. Three positive transgenic plants and two negative transgenic plants are selected and evaluated for WYMV resistance in T0 generation.
TaRx edited plants are generated with CRISPR-Cas9. Four edited plants for TaRx are derived and evaluated for WYMV resistance in T2 generation.
The mutants for TaRx are generated using EMS treatment. Three individual mutants are evaluated for WYMV resistance in M2 and M3 generation.

Authentication

All the transgenic and gene-edited plants were generated following the supervision of MARA PRC. The transgenic plants did not show any secondary effects except for the WYMV resistance enhancement. The off-target chance of CRIPSR-Cas9-induced TaRx gene edited plants was controlled by designing targets having single hit in the whole genome reference sequence.
